# Serum leptin levels are independently related to the incidence of ischemic heart disease in a prospective study of patients with type 2 diabetes

**DOI:** 10.1186/s12933-015-0208-1

**Published:** 2015-05-22

**Authors:** Camilla Vavruch, Toste Länne, Mats Fredrikson, Torbjörn Lindström, Carl Johan Östgren, Fredrik H Nystrom

**Affiliations:** Department of Medical and Health Sciences, Linköping University, SE 581 85 Linköping, Sweden; Department of Clinical and Experimental Medicine, Faculty of Health Sciences, Linköping University, Linköping, Sweden

**Keywords:** Blood pressure, Intima media thickness, Ischemic heart disease, Leptin, Obesity, Pulse wave velocity, Type 2 diabetes

## Abstract

**Background:**

New and clinically useful markers of cardiovascular risk are of essence in type 2 diabetes since ischemic heart disease is a major cause of death in these patients.

**Methods:**

We analyzed baseline data from 476 men and 244 women who participated in “Cardiovascular Risk factors in Patients with Diabetes -a Prospective study in Primary care” study. All participants had type 2 diabetes and were 55-66 years old at recruitment during year 2005 to 2008. Except for established traditional risk markers for vascular disease, we also estimated vascular complications non-invasively by performance of carotid-femoral pulse-wave velocity (PWV, with applanation-tonometry) and intima-media thickness of carotid arteries (IMT, with B-mode ultrasound). Patients were followed for incidence of ischemic heart disease mortality and morbidity until end of the year 2012, using the national Swedish Cause of Death and Hospitalization Registries.

**Results:**

During the follow-up period of a median of 6 years 47 men and 10 women died or were hospitalized for ischemic heart disease including myocardial infarction. Leptin levels were positively related to the hazard ratio (HR) in men (HR for each log 10 unit 4.9, CI 1.99 to 11.8) and women (HR 11.5, CI 1.47 to 89.7). Leptin predicted ischemic heart disease independently of age, HbA1c, BMI, systolic blood pressure and LDL-cholesterol/HDL-cholesterol ratio (men: HR 12.9 CI 3.2-53, women: HR 19.9, CI 1.2-327) This finding of increased risk related to high leptin levels was also statistically significant when carotid-femoral PWV and IMT were both added to the equations in men (hazard ratio 9.2 CI 2.1-41).

**Conclusions:**

Our data support the use of serum leptin in type 2 diabetes to add independent prognostic information in terms of ischemic heart disease when compared with traditional cardiovascular risk factors. In the men of the cohort this prognostic information was in addition also to data on IMT and PWV, two non-invasive measurements of the extent of vascular disease. The power to detect a similar relationship in women was less strong due to lower incidence of cardiovascular disease.

**Trial registration:**

ClinicalTrials.gov: NCT01049737.

## Background

New and clinically useful markers of risk are of essence in high risk states such as type 2 diabetes mellitus. The major cause of death in type 2 diabetes mellitus is cardiovascular disease. One of the strongest predictors of such manifestations of arteriosclerosis is the pulse wave velocity in aorta, which is a non-invasive measure of the degree of arterial calcification [1]. Yet another non-invasive estimation of the degree of vascular disease can be done by evaluating the thickness of the intima and media of carotid arteries by ultrasound [2]. Both these clinical physiological examinations are non-invasive and hence risk-free to perform. However, both these measures of risk require skilled personnel and the equipment needed also carries a considerable cost. Leptin is a hormone that is secreted by adipose tissue that regulates metabolism and reduces appetite. Levels of leptin in humans are log-linearly increased in relation to obesity [3] and lack of leptin induces severe obesity, demonstrating that leptin signaling is necessary for normal feedback control of body weight [4]. The feedback information to the central nervous system regarding adipose tissue size by leptin is influenced by so called leptin resistance [5] which likely is a reason for poor effect to reduce body weight with recombinant leptin in humans [6]. Potential adverse effects of this hormone includes increased activity of the sympathetic nervous system and of the adrenal glands to secrete corticosteroids [7] and leptin thus not only increases metabolic rate but it also elevates blood pressure according to experimental studies [8-11]. Leptin also has immune-modulatory effects and it has been suggested that this hormone links obesity with inflammation and hence with cardiovascular disease by such mechanisms [12-14].

Since leptin apparently interact with many of the features of cardiovascular risk, such as blood pressure and inflammation, it might be a useful marker of prognosis that gives information that is independent of a cluster of classical clinical risk factors. Thus we prospectively studied leptin levels in relation to ischemic heart disease in middle aged patients with type 2 diabetes mellitus. In summary we found that levels of leptin were positively related to the hazard ratio (HR) of ischemic heart disease in both men and women and this was independently of age, HbA1c, BMI, systolic blood pressure and LDL-cholesterol/HDL-cholesterol ratio.

## Methods

We analyzed baseline data from 244 women and 476 men who participated in a community-based cohort, CArdiovascular Risk factors in patients with DIabetes – a Prospective study in Primary care, acronym CARDIPP, in which serum leptin was measured at the baseline investigation. This prospective observational study was launched in November 2005 and recruitment of the participants was completed in 2008. The patients all had type 2 diabetes mellitus and were between 55–66 years old when they were consecutively recruited from 22 different primary health care centers in the counties of Östergötland and Jönköping, Sweden. Detailed information about the structure and results from CARDIPP has been described previously [15]. The centres were located in different demographic areas and differed in size. However, the model of treatment and care of diabetes was organized similarly and all the primary care centres adhered to the same national guidelines of diabetes care. The size of the cohort and age of participants was chosen to allow collection of endpoint data within 6 years, when assuming similar incidence of cardiovascular disease as in other cohorts of diabetes in Sweden [16].

### Anthropometric measurements

Nurses dedicated to treatment of diabetes at the primary health care centers measured height to the nearest cm and weight to the nearest 0.1 kg with the patients wearing light indoor clothing. Waist circumference was measured according to WHO’s recommendations with the patient standing, after a regular expiration, to the nearest cm, midway between the lowest rib and the iliac crest. A standardized medical history was taken that included medication, previous cardiovascular diseases and smoking habits.

### Laboratory analyses

Blood was drawn in the morning after a 10 hour over-night fast. Standard tests such as HbA1c, plasma glucose and serum lipids were analyzed according to routines at the primary health care centers. The laboratory analyses were performed at Department of Clinical Chemistry at the Linköping University Hospital. Levels of cholesterol, HDL and triglycerides were measured with enzymatic methodology and spectrophotometry, (Selectra E, Vital Scientific, Dieren, Netherlands). LDL was calculated by Friedewalds formula: LDL = cholesterol - HDL - 0.45 × fS/P-triglycerides. HbA1c was analyzed according to the Swedish Mono-S HPLC, all HbA1c values were converted to DCCT standard values using the formula: HbA1c DCCT = 0.923 × HbA1c (Mono-S) + 1.345 (R^2^ = 0.998).

The Milliplex® MAP Gut Hormone Panel (Merck Millipore, Billerica, MA, USA) designed for analysis with Luminex®-technique (Luminex, Austin, TX, USA) was used for analysis of leptin, in concordance with accompanying instructions. Total coefficient of variation (intra + inter assay) for leptin was 11%. The leptin levels were corrected for the inter-batch difference of control samples in the 10 batches in total used in the Luminex based analysis of the total cohort.

### Clinical physiological investigations

Determination of pulse PWV was done at the Department of Physiology, Linköping University Hospital, Linköping and at the County Hospital Ryhov, Jönköping. Aortic PWV was measured with applanation tonometry (SphygmoCor® system, model MM3, AtCor Medical, Sydney, Australia) over the carotid and femoral arteries. The aortic pulse wave transit times were measured by electrocardiogram-guided readings of the femoral arterial pulse waves, using the carotid arterial pulse wave as the reference site. The surface distances were estimated from the suprasternal notch to the carotid and femoral measurement sites, respectively. PWV was calculated by dividing the surface distance with the pulse wave transit time yielding m × s-1.

Intima-media thickness of the carotid arteries (IMT) were evaluated using B-mode ultrasound. A digital ultrasound system (ATL HDI 5000, Bothell, WA, USA) equipped with a broadband linear transducer (L12-5) was used for scanning the carotid artery in the longitudinal section. A 10 mm long section of the common carotid artery in the proximity of the carotid bulb was selected to obtain mean lumen diameter and far wall IMT. Three consecutive frozen images with focus on lumen-intima echo and media-adventitia echo of the far arterial wall were used. The digital B-mode images were subsequently transferred to a computer with dedicated software for off-line measurement of IMT (Artery Measurement System II, Image and Data Analysis, Gothenburg, Sweden). Calibration and subsequent measurement was performed by manually tracing a cursor along the leading edge of the intima-lumen echo of the near wall, leading edge of the lumen- intima echo and media-adventitia echo of the far wall. During analysis, the measurement window was hidden for the reader and values were saved in a text file. Mean values of IMT from both the right and the left sides were used in all analyses.

### Ischemic heart disease during follow-up

The participants were followed for occurrence of death or hospitalization for myocardial infarction or ischemic heart disease until December 31, 2012, by linkage with the Swedish Cause of Death and Inpatient registries (The National Board of Health and Welfare, Stockholm, Sweden) using their unique personal identification numbers.

### Ethics

All participants gave written informed consent prior to participating in the study. The study, which complied with the declaration of Helsinki, was approved by the Regional Ethical Review Board in Linköping, Sweden.

### Statistics

IBM SPSS statistics 21 and 22 (IBM corporation, Somers, NY, USA) were used for statistics. In the main statistical analyses, Cox regression (Proportional Hazard Model) was used. Pearson correlation coefficients were also calculated and statistical significance was defined as p < 0.05. Leptin levels were log-transformed in calculations since the distribution is skewed. Data were analyzed separately in men and women since relationship between leptin and obesity differs in genders [3]. The power of the study regarding an association between leptin and ischemic heart disease was greater than 99%, both for males and females.

## Results

Data on leptin in serum were successfully obtained in 246 women and 474 men in the cohort. There was strong a positive linear relationship between leptin levels and BMI (men: r = 0.631, women: r = 0.667, both p < 0.0001). Women had higher leptin levels (p < 0.0001) also when the comparison was corrected for BMI (p < 0.0001). Table 1 shows baseline characteristics in men and women analyzed separately. There were no statistically significant correlations between leptin and age, duration of diabetes or HbA1c levels in either gender (all p > 0.19 in men or women). In univariate analyses leptin levels correlated positively with PWV in men (r = 0.181, p < 0.0001) but not with IMT (r = 0.06, p = 0.2), and these findings were also apparent in women (leptin with PWV: r = 0.32, p = 0.049, leptin with IMT: r = -0.10, p = 0.10). Leptin levels were positively correlated with CRP (levels above 5 mg/l excluded, considered to be outliers) in men (r = 0.248, p < 0.0001, 72 excluded) and women (r = 0.187. p = 0.017, 69 excluded).

**Table 1:** **Baseline characteristics of the final cohort**

**Variable**	**Men (n = 476)**	**SD**	**Women (n = 244)**	**SD**
	**Mean level**		**Mean level**	
Age (years)	60.69	3.1	60.65	3.1
Diabetes duration (years)	7.1	6.36	7.4	5.7
Current smoker (%)	15.9		21.6	
BMI (kg/m^2^)	29.7	4.2	*31.0	5.4
Waist circumference (cm)	105.2	11	*103.0	13
HbA1c (mmol/mol)	53.0	12	52.8	12
HbA1c (%, Mono-S)	6.09	1.1	6.06	1.12
Total cholesterol (mmol/l)	4.62	.92	4.95	1.03
HDL cholesterol (mmol/l)	1.23	.32	*1.40	.35
LDL cholesterol (mmol/l)	2.62	.75	*2.77	.86
LDL/HDL ratio	2.22	.73	*2.07	.78
Triglycerides (mmol/l)	1.81	1.2	1.78	.88
Systolic BP (mmHg)	136.9	16	137.4	17
Diastolic BP (mmHg)	80.7	10	*78.5	9.9
Antihypertensives (number of drugs)	1.07	1.0	1.00	.93
Statin treatment (%)	53.0		58.0	
OAD (number of drugs)	.771	.75	.758	.70
Insulin treatment (%)	29.0		33.2	
PWV (m/s)	10.39	2.1	10.27	2.0
IMT (mm)	.755	.19	*0.702	.15
Leptin (ng/ml)	2561	2389	*7159	5654

*Denotes statistical difference between genders.

Abbreviations: BMI, body mass index; BP, blood pressure; HDL, high density lipoprotein; IMT, intima-media thickness of carotid arteries; LDL, low density lipoprotein; OAD, oral antidiabetic drugs; PWV, carotid-femoral pulse wave velocity.

Data was missing in 54 patients for duration of diabetes; 13 patients about smoking; 1 patient for BMI; 5 patients for waist circumferences; 10 patients for HbA1c; 23 patients for cholesterol; 3 patients for BP; 20 patients for IMT and in 59 patients for PWV.

Cox regression analyses were performed separately in men and women. No patients were lost to follow-up. During the follow-up period of a median of 6 years 47 men and 10 women died or were hospitalized for ischemic heart disease including myocardial infarction. Of these 57 incidents, 30 were due to angina, 21 of myocardial infarction and the remaining 6 were deaths due to ischemic heart disease. Serum leptin levels (crude) were positively related to the composite endpoint in men (hazard ratio for each log 10 unit of leptin 4.851, CI 1.987 to 11.84) and women (hazard ratio of 11.5 for each log 10 leptin unit, CI 1.468 to 89.73). When entering major known cardiovascular risk factors to the Cox regression leptin levels were positively related to ischemic heart disease in men, independently of age, HbA1c, BMI, systolic blood pressure and LDL-cholesterol/HDL-cholesterol ratio, smoking, and known duration of diabetes (Table 2a). This finding of increased risk for ischemic heart disease related to leptin levels was also statistically significant when carotid-femoral PWV and IMT of the carotid arteries were added to these equations in men (Table 2b), while in women the relationship was significantly related with the LDL/HDL ratio (Table 2d).

**Table 2:** **a-d Hazard ratios for ischemic heart disease during follow up**

**Variable**	**Hazard ratio**	**Lower CI**	**Higher CI**	**P value**
**Men**				
2a				
Age (years)	.987	.882	1.106	.828
Diabetes duration (years)	1.034	.994	1.076	.098
Smoking (current, former, never)	1.883	1.126	3.151	.016
BMI (kg/m^2^)	.913	.822	1.014	.089
Systolic blood pressure (mmHg)	1.030	1.010	1.049	.003
HbA1c (mmol/mol)	.999	.973	1.027	.958
LDL/HDL-ratio	1.260	.810	1.961	.305
Serum leptin (log 10 of ng/ml)	12.895	3.162	52.589	<0.001
2b				
Age (years)	.968	.861	1.089	.591
Diabetes duration (years)	1.034	.991	1.079	.127
Smoking (current, former, never)	1.570	.913	2.700	.103
BMI (kg/m^2^)	.946	.842	1.063	.349
Systolic blood pressure (mmHg)	1.024	1.003	1.046	.028
HbA1c (mmol/mol)	1.001	.971	1.032	.947
LDL/HDL-ratio	1.189	.737	1.919	.478
IMT (mm)	2.839	.721	11.180	.136
PWV (m/s)	.953	.800	1.134	.584
Serum leptin (log 10 of ng/ml)	9.219	2.088	40.705	.003
**Women**				
2c				
Age (years)	1.073	.861	1.337	.530
Diabetes duration (years)	1.060	.963	1.166	.231
Smoking (current, former, never)	1.190	.470	3.013	.713
BMI (kg/m^2^)	.995	.854	1.159	.949
Systolic blood pressure (mmHg)	.980	.932	1.030	.418
HbA1c (mmol/mol)	1.050	.992	1.110	.090
LDL/HDL-ratio	1.733	.789	3.804	.171
Leptin (log 10 of ng/ml)	19.9	1.205	327	.037
2d				
Age (years)	1.118	.831	1.506	.461
Diabetes duration (years)	1.08	.958	1.219	.205
Smoking (current, former, never)	1.18	.365	3.815	.783
BMI (kg/m^2^)	.934	.739	1.181	.570
Systolic blood pressure (mmHg)	1.035	.967	1.107	.321
HbA1c (mmol/mol)	1.06	.990	1.143	.094
LDL/HDL-ratio	3.85	1.091	13.6	.036
IMT (mm)	57.913	.603	5563.346	.081
PWV (m/s)	.515	.262	1.013	.055
Leptin (log 10 of ng/ml)	26.8	.432	1662	.118

Cox regression of ischemic heart disease incidence (Proportional Hazard Model) in relation to leptin levels and traditional risk factors, with (2b and 2d) or without (2a and 2c) addition of intima-media thickness of carotid arteries and carotid-femoral pulse wave velocity. Table 2a and b are data for the men, 2c and 2d show data for the women.

Abbreviations: BMI, body mass index; HDL, high density lipoprotein; IMT, intima-media thickness of carotid arteries; LDL, low density lipoprotein; PWV, carotid-femoral pulse wave velocity.

**Figure 1:**
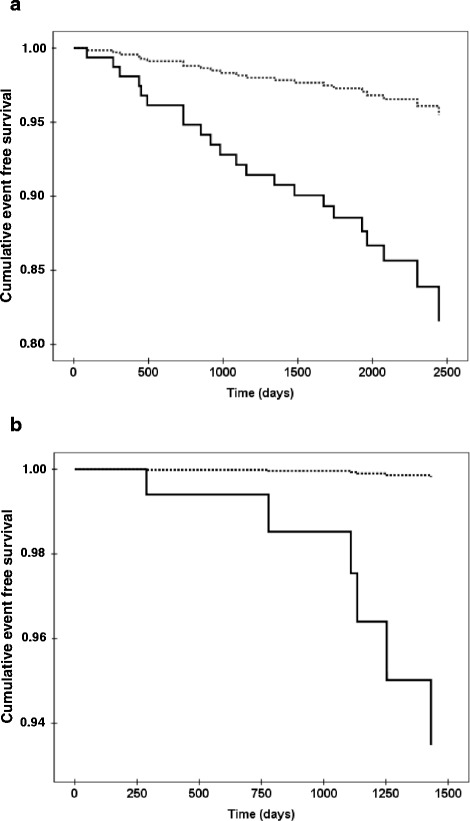
Cox regression analyses of event free survival in relation to leptin levels. The dashed line represents the lower tertile and solid line the higher tertile of leptin levels in men **(a)** hazard ratio 9.219, confidence interval from 2.088 to 40.705, p = 0.003 and women **(b)** hazard ratio 26.8 confidence interval from 0.432 to 1662, p = 0.118. Data are adjusted for age, known duration of diabetes, HbA1c, systolic blood pressure, smoking, LDL/HDL- ratio, BMI, carotid-femoral PWV and carotid IMT.

Figure 1a and b show Cox regression analyses of levels of leptin in the lowest and highest tertiles in men and women, after correction for the risk factors as in Table 2b and d, respectively. When CRP was added to the analyses in Table 2b and d leptin levels were still significantly related to ischemic heart disease in men while CRP was not (leptin: p = 0.005, CRP: p = 0.96), however, neither of these markers were independently related with ischemic heart disease incidence in the corresponding analyses in women (leptin: p = 0.68, CRP: p = 0.25).

## Discussion

We found that high levels of leptin were related to increased risks of ischemic heart disease independently of traditional risk factors in men and that this prognostic information also remained significant when carotid IMT and aortic PWV were both included in the Cox regression. Importantly these analyses included BMI as a measure of obesity and this implies that leptin gave additional information independently of the degree of adiposity. This is in line with the idea that leptin resistance, an insensitivity to feedback signaling of adipose stores to the CNS, is linked with increased risk for ischemic heart disease in patients [17]. However, since our study did not include an intervention aimed to affect leptin levels, this does not provide definite proof of causality. Elevated levels of CRP have been suggested to participate in induction of leptin resistance by direct interaction with the leptin molecule [18]. The positive correlation between leptin and CRP in both men and in women in our study is in line with such a theory. Also leptin is a regulator of the sympathetic nervous system with effects that, according to experimental studies, include increases of metabolic rate, angiogenesis and arterial tone [9]. This could theoretically have impact on ischemic heart disease incidence that goes beyond many traditional markers of cardiovascular risk, including BMI [19] which is in line with our findings in men with type 2 diabetes mellitus. High circulating leptin levels have earlier been reported to be associated with diabetes incidence and also with development of other components of the metabolic syndrome [20]. However, leptin levels were not found to be related to cardiovascular morbidity and mortality in a study of non-diabetic elderly people [21].

In a study of patients with type 2 diabetes mellitus by Brennan et al. leptin levels were reported not to be related to cardiovascular disease in 1194 women that were followed for 12 years [22]. This finding was in contrast to our data which do support prognostic use of leptin in serum when added to traditionally used risk factors, to estimate risk for ischemic heart disease in women. However, statistical significance was lost in the women in our trial when IMT and PWV were included in the equations. It is possible that less efficient treatment of complications in diabetes might have obscured specific actions of leptin, not acting through traditional risk factors, in the study by Brennan et al., since data were analyzed during 12 years up to the year 2002 [22], i.e. ending 3 years before we made our baseline investigation. In line with this LDL-cholesterol levels were 3.4-3.7 mmol/l across quintiles of leptin in the study by Brennan et al. while corresponding mean value was 2.77 in the women in the study presented herein. This is suggestive of more efficient lipid lowering therapy in our study which has been a trend in treatment of type 2 diabetes mellitus in general during the last decade [23]. It is also possible that age of participants is crucial in analyses of the risks related to leptin since at higher age obesity is not strongly linked with morbidity and mortality. Indeed, the term “obesity paradox” has been applied to the phenomenon that moderate obesity might even be beneficial in older age [24]. Indeed, better prognosis in obese as compared with leaner subjects in patients with diabetes > 65 years of age has been demonstrated [25] and patients in the study by Brennan et al. became older during follow up [22] than those in our cohort.

In males the relationship between leptin and IHD was independent of traditional risk factors which was in line with earlier trials in men with high risk but not necessarily restricted to type 2 diabetes [26-29]. However, a study of patients which also included a large proportion of patients with diabetes and renal impairment did not find an independent relationship with cardiovascular events of leptin when analyzed among many other cytokines [30]. We acknowledge that we had no information about cytokines such as adiponectin or macrophage migration inhibitory factor as in the paper by Schöttker et al. [30] but we did find leptin to be independently related to ischemic heart disease also in combination with two specific measures of arteriosclerosis, IMT and PWV. This might seem contradictory if leptin indeed mainly relates to arteriosclerotic disease *per se*. Potential explanation could be that PWV and IMT do not capture all information about true burden of arteriosclerosis. Another potential explanation is that although IMT and PWV together carry much information about arteriosclerosis in large vessels, we do not gain much knowledge from these measures of risk for plaque rupture which is the main cause of acute ischemic heart disease and which is to a large extent dependent on whether a plaque is vulnerable or stable [31]. Indeed, leptin has been found to be related to endothelial dysfunction in diabetes [32].

Strengths in our study include use of registry data with high quality and in terms of mortality and morbidity incidence no lost to follow-up. Weaknesses include a rather short follow-up time and consequently low rates of mortality and morbidity. However the relatively short follow-up period suggests clinical relevance insofar that patients were indeed being cared for in a manner that is in accordance with current standards. This was confirmed by frequent treatment with statins and antihypertensive medications, and levels of HbA1c and blood pressures were also generally close to targets recommended by guidelines. Indeed, it is possible that our finding of an association between circulating leptin and risk for ischemic heart disease was statistically significant after correction for traditional risk factors in men and women was made possible by reasonably good control of glycaemia, blood pressure and cholesterol that increased the chance to detect the risk related leptin, that might act through other mechanisms than mediated by the traditional risk factors that are known to the provider of care. The positive relationship between serum leptin levels and CRP, confirmed in our study, is an example of such a risk factor that is not targeted in routine clinic care, as of yet.

## Conclusions

This prospective observational data support the use of serum leptin for estimation of risk for ischemic heart disease independently of traditional risk factors. In the men serum leptin also provided information about risk that was independent of two strong non-invasive markers of cardiovascular disease, carotid IMT and carotid-femoral PWV. The results of our study support further assessment in larger cohorts of the role of measuring leptin levels as a predictive marker if ischemic heart disease in the care of patients with type 2 diabetes.

## Abbreviations

BMI: Body-mass index
BP: Blood pressure
CARDIPP: CArdiovascular Risk factors in patients with DIabetes – a Prospective study in Primary care
CRP: C-reactive protein
IHD: Ischemic heart disease
HR: Hazard ratio
IMT: Intima-media thickness
OAD: Oral antidiabetic drug
PWV: Pulse-wave velocity
WHO: World Health Organization
